# Monthly Summary

**Published:** 1897-06

**Authors:** 


### Monthly Summary.

DENTAL OPERATIONS DURING PREGNANCY.—By Dr. H. J. Custer, Johnstown.—Several years ago a young married lady of lymphatic temperament presented herself in order to have a superior lateral incisor filled with gold. Nothing was said regarding her condition, but circumstances indicated the first weeks of pregnancy, and a cement filling was accordingly advised.

The patient insisted on a gold filling, which was finally agreed on. The cutting of the sensitive dentine apparently caused but little pain, in fact, the several steps of the operation were accomplished with but little discomfort till a finishing strip was introduced, when the first and only rasp caused a momentary muscular paroxysm. After a few moments' interval the filling was completed by the aid of disks, and the lady left the office without much depression and well satisfied with the operation. However, the second day after the operation uterin contractions were established, and after several days of rather serious illness an abortion of about the tenth week was completed.—*Welch's Monthly.*

---

SWAGING PLATES.—By J. Y. Crawford.—I recommend the recent developments in swaging metal plates over a plaster die; it can accomplish results that are perfectly marvelous. Nothing can proximate a lower plate of aluminum, swaged over a plaster die, with rubber attachments. It goes in like a wafer, and there will be no ulcerative absorption in the lower jaw. If the teeth are imperfectly occluded, the lower plate moves and slips about, and hits the mucous membrane here and there till the tissues become hypertrophied, and finally there remains only a soft bed for the plate to rest on. But there is less absorption under an aluminum plate swaged between two metal surfaces and driven home on a plaster die; it fits so beautifully you can bring out the rugæ in an upper plate. You have all seen those soft, mushy jaws, where all the anterior part is like a second tongue; but you will have no more of that if you will adopt the swaged aluminum. I earnestly recommend the process of swaging metal plates over the plaster die, and assure you you need not break the model.— *Welch's Monthly.*

---

A LIGHT ARTIFICIAL DENTURE.—By Dr. W. H. Todd, Columbus, Ohio.—After you have carved and prepared the plaster model as the mouth would indicate, it should be painted with a thick shellac varnish and covered, where you want the plate, with Japanese lead (lead foil that comes in chests of tea). Be sure to have the foil burnished on the model, so that every inequality is shown. Now get the articulation as usual. If you wish to try in, take a piece of hard base plate and cut it short of the alveolar ridge, so that when the teeth are set up it can be cut out, leaving the teeth standing. Mount the teeth and try in, if all right cut out the hard base plate as close to the pins of the teeth as possible, replacing the base plate with a plate of Japanese lead, using two or three pieces, according to thickness of plate required, smoothing up next to the teeth, also the rim, with wax, just as you want the plate when finished. Then take another piece of Japanese lead and cover the entire palatin portion and rim of plate to the teeth, always burnishing each piece down close.

The last piece cover with shellac varnish so that it will adhere to the other part of the flask, then flask up. Warm before separating; take out the middle pieces of foil and wash out with hot water. Use a piece of chamois skin and a little mercury, and proceed to polish the foil in both halves of the flask till it is as bright and smooth as glass, then you are ready to pack, using the toughest rubber you can buy.

When you take the plate out, you will have nothing to do but trim the edges and brush on the lathe, the result being the lightest, toughest and thinnest plate made.—*Ohio Dental Journal.*

SPACE IN FILLING.—Dr. Arthur B. Freeman: Dr. Brophy says that in filling proximal cavities, wedges should be placed between the teeth for several days. That occurs to me as being a mistake. When I entered the profession, some sixteen years ago, this was a common practice, and for a time I indulged in this method myself, but have discarded it for many years, believing that it is a mistake to subject patients to the soreness which necessarily follows wedging. The pain of introducing a filling when a tooth is in this tender condition is terrific, to say nothing of the injury often done to the process and other tissues. Dr. Brophy did not place any limitations on the statement he made, so I took it for granted that he wedged for several days, and followed immediately with the filling.

Dr. Truman W. Brophy: I did say that who ever made a critical examination of the proximal surfaces of teeth must secure space, and I am not one of those who believe in adjusting appliances and rapidly crowding the teeth apart. I have seen too many bad results from that kind of treatment. I am satisfied it is not possible in many cases to make a good proximal filling without securing space. Many times where caries is extensive the teeth will interlock, one will drop into the other. If you apply a separator between teeth of this character and attempt to crowd them apart, you subject the patient to more pain than if you were to use wax tape and pry them apart. You may take two or three days to do it. If you do that it will not cause any discomfort, whereas, if you resort to immediate separation you will injure the parts and the injury cannot be overcome by the lapse of time.—*Dental Review.*

---

ABSORBED RIDGE.—Where most of the alveolar ridge has been absorbed—especially the lower—it is often difficult to keep the plate in place, but a firm adhesion may be obtained by the following method:

After your plaster model is nearly dry, mix some plaster very thin, and with a fine camel's-hair pencil, build up a half round ridge, about the width of an ordinary knitting needle, all along the highest part of your model, or what is left of the original ridge, leaving off about a quarter of an inch from the end of the previously marked-off plate, on both sides, or the object will be defeated. Never wet the model, or your plaster will run where it is not wanted, while, when dry, you can guide your plaster with the pencil and build up a neat and even ridge; a very important point in taking the model from the molding sand afterward. The work should be performed neatly and rapidly, adding a little material at a time, till the ridge is finished to your satisfaction; when finally your plate is struck up, and while trying it in, you will be surprised to find how firmly it will adhere, by means of this narrow and continuous air-chamber.

The same process will hold good in rubber plates, but the model should be more thoroughly dried, to prevent the sticky base-plate from injuring the ridge. For troublesome shallow plates, already in the mouth, we would suggest the forming of a hollow ridge by means of an engine bur. We have used this process for lo! these many years, and have always found it a success in even the most desperate cases.—J. DIENELT, in *Office and Laboratory.*

---

AN IMPRESSION AND ITS TESTS.—By C. A. Devin.—A perfect impression must have fullness, smoothness and sharpness. It should cover a little more surface all around than the finished base-plate; should faithfully reproduce all the finest lines, and should be as smooth as the mucous membrane. The sharpness is required not only to attain the best adhesion, but to counteract as far as possible the blunting effect of cast varnish. Smoothness is necessary to the health of the surfaces against which the plate rests. A full impression adheres to the part it covers, either because the air is excluded from under it, or on account of alveolar undercuts, or because by being left in too long it has absorbed the moisture from the mucus membrane. The latter effect is generally caused by ignorance or carelessness, and usually injures the mouth. The resistance which occurs from pressure of the atmosphere against the under surface of the cup, showing that the air is excluded above, can always be relied on as the certain test of a good impression. The resistance which is offered by an undercut can easily be distinguished from that of the atmosphere, and is nearly always felt after the latter has been overcome. Of course it is admitted that an impression which drops when touched or is detached with very little force may be so modified as to produce a plate with fairly good adhesion; but such an impression cannot be considered reliable, and in difficult ones it is almost wholly worthless.—*Dental Journal.*

---

EUCAINE.—By Dr. Watkins.—Eucaine comes in the same form as hydrochlorate of cocaine, and requires to be prepared in distilled water, then boiled. You set it in a tin basin of water and boil it. Then you have it pure and free from germicides. Wipe the gum clean with a weak solution of carbolic acid and you will be introducing no poison, and will create no inflammation. Eucaine, unlike cocaine, will keep indefinitely; it will keep till it is used up. You can inject a five or ten per. cent. solution into the gum each side of the root; only a short time is required for its effect; from three to five or eight minims is a sufficient quantity, and you can extract the tooth usually with no pain. It has worked better in my hands than any other preparation that I have tried. The other day I had a case to prepare for an upper and under set, nothing but roots to remove. I made two injections, first from one wisdom tooth to the central, then extracted all the roots on that side; then injected from the other wisdom tooth to the central, and extracted on the other side. There was no pain except in the removal of each of the wisdom teeth. Perhaps I did not introduce quite enough of the remedy, or did not leave it long enough at that point. As to the rest, the patient said it was without pain. I have used this eucain in quite a number of cases, and it works nicely, seems to be harmless, and leaves no sore mouth afterward.—*International.*

CARBOLATE OF IODIN.—By E. G. Betty.—Its prepara- is at once simple and ready and should be done just before using. To about two or three ounces of distilled or filtered water, which has also been boiled, but reduced to 100 degrees F., add first ten or twelve minims of Calvert's crystallized carbolic acid No. 1, then the same quantity official tincture iodin. Stir this up by filling the syringe bulb and injecting back into the glass. The muddy color of the iodin will disappear, with an apparent evolution of gas. A peculiar odor is at once perceived, entirely different from that of either of the principals. When gingival inflammation is present, use as hot as the patient can endure; and for all purposes it is well to use it quite warm.

I have found this far superior to listerin, boracic acid and all that class.—*Review.*

---

GALVANIC ACTION IN THE MOUTH.—I have often heard of galvanic action of metals in the mouth, but the most complete battery I ever heard of, I produced in my own mouth. It may be interesting to others to have a history of the case.

My first molar was a wreck, so I took an aluminum crown, cut top off, and slipped the band to place—packed asbestos in bottom of cavity and then contoured it up nicely with alloy. The tooth looked well and the articulation which I sought was perfect. The adjacent bicuspids were gold crowned, as well as the first molar and second bicuspid below.

I thought it a success until I came to close my mouth, and then I found that I had a perfect galvanic battery. All was well when my mouth was open or when firmly closed—but when the teeth were nearly closed I received the shock. I endured it for an hour or two and then I slipped the band off and all was well again, except that the second bicuspid crown had changed in color to perfect aluminum, and was with difficulty restored.

The aluminum was the exciting cause, but the query is, would the effect have been the same had I left out the alloy and used the aluminum crown in the usual way?

DIFFICULT PARTIAL IMPRESSIONS.—By Homer Heberling, D. D. S., Lehighton, Penn.—Several weeks ago a lady presented, desiring a partial upper denture. An examination of the oral cavity revealed a V-shaped arch with several sound teeth, posteriorly and anteriorly.

Wherever possible, I prefer taking plaster impressions in partial caaes; therefore, I proceeded in the usual manner to get a plaster impression. The teeth were at unfavorable angles, seriously interfering with the work. After several unsuccessful attempts and spending considerable time in trying to patch together broken pieces which were more intricate than a Chinese puzzle, I hit upon the following method:

That portion of the plaster which came out intact, viz: the palatal surface, festooning teeth lingually, and partially covering the ridge where teeth were absent, I replaced carefully in the impression-tray, flowing *very* soft plaster where the first impression had been imperfect. I next pressed the tray and plaster quickly into the arch, retaining it in position until the new plaster had set in. The cup was then easily removed, the new plaster fractured cleanly and in large pieces, some, however, remaining cemented to the original impression, making it a simple task to reunite the whole. The result was a perfect model of all the teeth, ridge palatal surface and condyles, from which I made a well-fitting plate.—*Items of Interest.*

---

MANY men are everlastingly preaching cleanliness to patients and instructing them to brush their teeth when they themselves are not clean. This is a mistake.—Ira B. Crissman, in *Review.*

---

TO PREVENT RUST.—If iron, steel, nickel or copper instruments are dipped in alcohol containing a little borate, carbonate, bicarbonate or benzoate of soda, they will not tarnish.—*Lancet-Clinic.*

FUNCTION OF THE UPPER THIRD MOLAR.—As this tooth is the only one of the dental caries which presents the typal three basal cones which enter into the construction of the typical upper molar, some explanation is frequently demanded as to why this should be the smallest of the molar teeth, and why the small molar does not approximate the bicuspid teeth. It may be possible that the following explanation has been published before, but I have never seen nor heard it and therefore present it.

It will be noted that the lower jaw is a lever of the third class, the power being placed between the fulcrum and the weight; the fulcrum, the temporo-maxiliary articulation; the power, the direct muscles moving the mandible, the masseter, and temporal; the weight is represented by the resistance of substances in process of being incised or crushed between the teeth.

Being a lever of the third class, it follows that, with a given amount of power, the resistance it can overcome is governed by the distance of this resistance from the fulcrum; that is, the molar teeth exert more force upon food masses placed between them than do the incisors. This third molar being nearest the fulcrum, exerts more pressure upon bodies against which it is pressed than any tooth anterior to it. It is therefore by anatomical position designed for the application of great force. Its root or roots being curved backward, instead of being straight, will produce a greater diffusion of force received upon the crown than were the roots straight; this is also true of the lower third molars. The mechanical element in original root formation is, for the present, left out of consideration.

It is seen that in the occlusion of the upper third with the lower third molar, the conical palatal cusp of the upper tooth is in antagonism with the broad occlusion surface of the lower third molar. The lower molar, therefore, is a base upon which a body is to rest which is to be acted upon by a heavy conical antagonist. The usual functions of the teeth, incising and crushing, are clearly not called for, as the structures named are not of fit design for such a purpose. Any one may test, by simple experiment, that the mechanical design described is one fitted best for the breaking, not crushing, of hard, brittle bodies; the firm base, conical tool, and great force all point to this. The articles entering into a dietary which call for these forces are nuts which have hard, resistant shells; *ergo*, the function of the third molars is the crushing of nuts.

It is offered as a suggestive datum to those who are interested in the philosophy of evolution, that the crushing of nuts, or for that matter any hard and brittle foods, is no longer called for in the maxiliary work of the civilized man, and physiological disuse is a corollary. On the other hand, it is not to be forgotten that the buccal cusps of these teeth have their function, which, by the same reasoning, are adapted to the masticating of tough meats and vegetable fiber.—H. H. Burchard, *Dental Cosmos.*

---

TO DIAGNOSE PULPITIS.—Since the recitation of cases seems to be in order, I will refer to one that I have recently had. It relates to the subject of pulpitis. The question has risen as to the difficulty of diagnosing these cases. It is difficult sometimes to locate the pain. Patients frequently refer it to some remote point. A lady came to my office early in July saying that she was having trouble on the right side, pointing to the interproximate space between the second bicuspid and first molar. She said the pain was located there. I made a careful examination and could find no cavity. The pain was not severe and I told her it was probably only temporary. While I was absent from the city she had a recurrence of the pain and had the tooth examined by some other dentist, and he was under the impression that there was a cavity in the first molar. However, she did not have anything done, and came to my office one day after I had returned. She came in the midst of excitement and began to take me to task for overlooking a cavity and allowing her to suffer this pain. She pointed out the tooth which she thought was giving the trouble. She pointed definitely to the second bicuspid. I examined it, and could find no cavity in either the first molar or second bicuspid.

My method of diagnosing these cases is this: Instead of cold I used heat. I take a cone of heated gutta-percha, which is tenacious and better than a hot instrument. It will cling to the surface of the tooth. I applied this to the second bicuspid and she flinched. I said: Is that the character of pain you have been suffering?" She replied, "No." I then went from one tooth to the other until I came back to the third molar, which had a large oxyphosphate filling in it. When I applied the heat to it she flinched again, and exclaimed, "There, that's the tooth. I told you all the time it was that one," and she put her finger on the second bicuspid. I drilled through the oxyphosphate filling and struck a case of pulpitis. I destroyed the pulp and this was the end of the trouble. This is another instance illustrating the fact that we can not take a patient's word as to what particular tooth is giving trouble. We must find it out ourselves. The rule I follow is to test one tooth after another. The patient will flinch when a living tooth is touched, but it will not be the same character of pain that they have been suffering. It is momentary and passes away. But the moment you strike the right tooth they will say, "That is the kind of pain I have been suffering."—C. N. Johnson, *Dental Review*.

---

TO RELIEVE SENSITIVENESS AFTER GRINDING A TOOTH.—Apply a saturated solution of carbonate of potassium and glycerin.—F. L. Platt, in *Dental Digest*.

